# Digital epidemiology and global health security; an interdisciplinary conversation

**DOI:** 10.1186/s40504-019-0091-8

**Published:** 2019-03-19

**Authors:** Tim Eckmanns, Henning Füller, Stephen L. Roberts

**Affiliations:** 1 Robert Koch Institute, Berlin, Germany; 2 Geography Department, Humboldt-Universität zu Berlin, Berlin, Germany; 3 Department of Health Policy, London School of Economics (LSE), London, UK

**Keywords:** Digital epidemiology, Big data, Algorithms, Infectious disease, Global health, Security

## Abstract

Contemporary infectious disease surveillance systems aim to employ the speed and scope of big data in an attempt to provide global health security. Both shifts - the perception of health problems through the framework of global health security and the corresponding technological approaches – imply epistemological changes, methodological ambivalences as well as manifold societal effects. Bringing current findings from social sciences and public health praxis into a dialogue, this conversation style contribution points out several broader implications of changing disease surveillance. The conversation covers epidemiological issues such as the shift from expert knowledge to algorithmic knowledge, the securitization of global health, and the construction of new kinds of threats. Those developments are detailed and discussed in their impacts for health provision in a broader sense.

## Introduction

The term digital epidemiology is in this special compilation defined by Marcel Salathe as epidemiology that uses data that was generated outside the public health system, i.e. with data that was not generated with the primary purpose of doing epidemiology (Salathe 2018). Arguably a narrow definition, we will use this conceptualization as the starting point for our conversation. The so defined digital epidemiology promises faster detection of disease outbreaks and improved surveillance as well as reduction in administrative and financial burden**,** among other things. At hand in the following conversation is less the question if those promises are kept. Instead we are interested to reflect epistemological/methodological, ethical/legal, social/political, and organizational aspects and implications corresponding to the promise of digital epidemiology. What will be the relationship of traditional and digital epidemiology? Will a possible change influence the scope of Public Health and Global Health? Tim Eckmanns, Henning Füller and Stephen Roberts discuss political implications of digital epidemiology.

## Tim

Digital infectious disease early detection systems such as the ProMed-mail, Global Public Health Intelligence Network (GPHIN), HealthMap, the now closed Google Flu Trends or the syndromic surveillance system ESSENCE are central elements of global public health surveillance.

However, with increasingly digitalized (algorithmic) global public health surveillance systems and related data-driven epidemiological analyses (e.g., Digital Epidemiology and other research methodologies), there seem to emerge epistemological shifts, as well as methodological ambivalences and diverse social and political effects.

You, Henning and Stephen, both work from a social (or rather political) science perspective on the societal implications of Digital Epidemiology, which is shaped by multiple imperatives, e.g., of ‘global health security’ as well the potentials of big data.

## Stephen

Over the past two decades, I would argue, we have seen an unleashing of the algorithm across practices of health security and surveillance. Algorithmically-guided infectious disease surveillance systems have proliferated across global health geographies, seemingly in response to a series of interconnected and complex transformations within global health governance (GHG), as well as the practice of international relations and international security. We have seen the rise of a seeming ‘epidemic of epidemics’ from the late twentieth century onward, including the emergence of HIV-AIDS, novel strains of avian and swine influenza, SARS, Ebola, MERS, the Zika virus, and the re-emergence of cholera, polio and multi-drug resistant Tuberculosis across low and middle income countries (LMICs) clinical and public health surveillance practices with their routinised processes of data collection, analysis, and dissemination from national health institutes have increasingly fallen out of pace with the capacity to timely identify the globalised spread of novel and re-emergent pathogens.

Correspondingly, the rise of the digital era, resultant from technological interconnectivity and innovation, has generated infinite, voluminous and diverse data at a rate never feasible in history. Between 2016 and 2018, 2.5 quintillion bytes of data have been produced continually, every day in the mere span 24 months (IBM 2018). Celebrated for the capacity to connect the operational ‘dots’ between these seemingly unintelligible and largely unstructured streams of data in the surveillance and identification of infectious disease outbreaks, the algorithm has emerged as salient and novel technology of security in the pre-emption of pandemic threats in the twenty-first century.

To firstly illustrate this shift, in late November 2002, the Global Public Health Intelligence Network (GPHIN), a semi-automated online health surveillance system, which piloted the use of retrieval algorithms to filter international media sources, identified the early reporting of a form of atypical pneumonia circulating in Guangdong Province, China. The ‘algorithmic gaze’ of GPHIN identified the origins of the severe acute respiratory syndrome (SARS) in advance of 3 months of traditional public health and governance authorities. More than a decade following the rapid spread of SARS, HealthMap, an online health surveillance system, identified again, via algorithmic processing of digital data streams, the emergence of a mysterious hemorrhagic fever occurring in Macenta, Guinea. HealthMap critically captured and presented strategic epidemic intelligence detailing the emergence of the Ebola Virus Disease (EVD) on 14 March 2014, 9 days in advance of the official notification of outbreak by the Guinean health authorities.

### Digital epidemiology - from expert knowledge to ‘knowledge without truth’

The increasing integration of algorithmically-driven infectious disease surveillance systems contemporary logics of health security are critical and significant for a number of reasons (Roberts and Elbe 2017). First, reflective of a growing recourse to the harnessing of novel information sources to contain pandemic illness, the WHO, via the revision of the International Health Regulations (2005), has clearly authorised the collection, assessment and utilisation of non-governmental sources of epidemic intelligence and data (Article 9.1), without prior clearance of member-states. (World Health Organization 2008) In this regard, the algorithm emerges a new purveyor of varied, voluminous and expedited data sources to be leveraged in the risk assessment of future infectious disease threats. Epistemically, what we can see is how the centralisation of the algorithm within security technologies such as digital disease surveillance systems re-contour previous relations and understandings of knowledge production, the practice of surveillance and the regulation of pandemic risk. The cultivation of knowledge to address the contingent within past ‘regimes of truth’ were largely sustained, as illustrated by Foucault by the ‘avalanche of statistical numbers’ (Hacking 1982). Increasingly however, within these contemporary security technologies, the 3 Vs of Big Data (volume, variety, and velocity) are now being mined, scanned, and reassembled via algorithmic processing of data to produce findings and alerts on the next pandemic. Information and ‘truths’ about the physical world and the contingent threat of infectious disease are increasingly extracted in the forms of signals and signs of the realm of the digital, and no longer solely generated from statistical processes via human analysis.

Furthermore, as the conceptual work of Antoinette Rouvroy (2011, 2013, 2015), has demonstrated, algorithms have emerged within health surveillance technologies as purely ‘rational’ or ‘objective’ instruments of forecasting, indifferent to the causes of phenomena and seeking only to accrue maximal reservoirs of data to address that which constitutes the contingent or the uncertain. What this means therefore is information and knowledge generated by these algorithmic techniques now appear to bypass the traditions of human assessment, analysis, hypothesis, testing and trial which were essential to the statistical calculation of the contingent. Rouvroy has referred to this dissemination of this new form of understanding future-situated uncertainty as “knowledge without truth”, represented in the context of this discussion by disease tracking systems including GPHIN and HealthMap, which have, with upward intensity sought to apprehend infinitely expanding data sources through an intensified recourse to algorithmic-suffused disease surveillance. What is absolutely vital to emphasise here is that amid the widespread deployment of big data analytics and increasingly sophisticated algorithms for tracing the next outbreak, little critical assessment has been formulated by global health security theorists and practitioners on the ramifications ‘digital’ turn of health surveillance and the implications of big data and algorithmic surveillance practices on individuals, populations and states.

Thus, these continued shifts towards employing advanced algorithms to make sense of unprecedented amounts of information (Leese 2014), across practices of contemporary disease surveillance must be continually matched with equally robust interrogations of the unforeseen or unprecedented implications of *securitization by algorithms* in the realms of ethics, law, politics and society.

## Henning

Thanks, Stephen for underlining the function of algorithms in current approaches of disease surveillance in global health policies. In addition, I pose that the rationale of an ‘emerging diseases world view’ (King 2002) is similarly influential for public health surveillance on a domestic scale. Especially in the US, systems of syndromic surveillance have been explicitly employed to answer the challenges of the ‘next pandemic’ with a new algorithmic form of public health monitoring. Going a bit into the details of one specific example of Syndromic surveillance, I want to illustrate the problem of “knowledge without truth” Stephen mentioned above. The argument is that those systems ‘call back’ in several ways, influencing both truth claims and practices of public health provision. My empirical example is a study on the use of the “Electronic Surveillance System for the Early Notification of Community-based Epidemics” (ESSENCE) in the U.S. National Capitol Region, an application of syndromic surveillance that received considerable attention as a pilot project (Füller 2018).

Technically the ESSENCE system provides the server infrastructure to draw together diverse data-sources that are considered indicative for public health. Its ‘syndromic’ approach consists in the integration of several so called surrogate data, signals of diseases or public health problems generated before a confirmed medical diagnosis (Velasco et al. 2014). In the case of ESSENCE, such surrogates are for example emergency department chief complaints, daily over-the-counter sales of the two big pharmacy chains *CVS and Rite Aid,* reports on absenteeism data gathered from public schools and others. ESSENCE claims to provide an unmatched situational awareness partly due to the near real-time nature of those data (collected and reported at least daily). Given the amount and unstructured nature of this data, the system employs algorithms to continually search the gathered data-stream for unusual patterns and a GUI to visualize and map resulting alerts. If there is an unusual coocurrence of for example the sale of headache pills and school absenteeism in a region, the system will flag out a warning. Importantly, the base for this pattern recognition are at no times diagnosed health problems but assumptions generated through the association of different data sets. Eventually the system promises to automatically provide an early notification of any unusual public health event before it has been medically diagnosed (Fearnley 2008).

The turn towards infection control and surveillance in public health and the introduction of syndromic surveillance systems have both been contested early on and from several vantage points (Reingold 2003). The focus here is to point out the performative character of technologies and their related practices in altering the goals and modes of public health provision.

My argument centers on the fact that the system is constantly producing health related truth claims. Whether it is just quietly monitoring – as it does most of the time – or in the rare cases that it is flagging out a public health emergency, the system claims a certain truth about the health of the monitored population. In both cases, the algorithmically produced knowledge becomes performative in different ways. Both forms of truth claims illustrate the ‘knowledge without truth’ problematic Stephen already mentioned.

On the one hand, those systems introduce a new expectation and a demand to constantly assure the normal state of affairs. New technologies of surveillance are employed to be able to illustrate an absence, to be able to constantly assure that there is nothing to worry about, as Kezia Barker argues (Barker 2014). In order to be aware of unusual events, resources, work and infrastructure are invested to extensively monitor the routine state of public health. But this additionally generated knowledge does not provide a qualified, actionable truth about the state of public health. Trying to see short-term events, those systems measure against the baseline of the ‘normal’. In its usually quiet mode of monitoring, the systems make the implicit claim about a ‘normal’, ‘well functioning’, ‘unproblematic’ state of public health, ignoring any long-term and structural health issues.

On the other hand, In the case of actually flagging out an incidence, automated monitoring systems such as ESSENCE are problematic in their rendering of disassociated facts into medical truths. The threat of an emerging public health event is especially burdening for the executive branch of the local state. Decision-makers are pressured to act early, at best before the expected cascading of an infection gets out of control. This expectation makes it tempting to base a decision on the syndromic signals as they are readily available and - through the included mapping tool - often clearly localized. While those signals are explicitly handled as an additional but clearly undiagnosed source of information among epidemiologists and public health experts, for the executive branch they have a tempting appeal of providing a near real-time situational awareness and as such an actionable grasp on the emerging public health event. Importantly, using ESSENCE as a base for decision-making approaches the signal as if it was an authoritative medical fact instead of just an indicator for the clustering of certain syndromes. The danger of misinterpretation as the algorithmically generated knowledge travels contexts may result in wrongly employed public health interventions with negative social effects. Besides the problem of false positives prevalent to those systems (Fearnley 2008) the system always suggests a spatialized source of the problem that may or may not be medically justified. Employing public health interventions based on those seemingly objective and localized realities can easily mean the wrong allocation of scarce resources and attention or effect an unjustified stigmatization of a ‘problematic’ area.

## Tim

From my perspective, as a medical infectious epidemiologist and public health expert who advises on the development of new surveillance systems and who constantly needs to be aware of their effectiveness as well as the consequences of their use, Stephen and Henning’s analyses offer extremely important contributions on how to think about and evaluate increasingly digitized health- and infectious disease control. To add to this, I would, in the following, like to make a few further comments about the epistemic and political aspects of the digitization of infection control. In particular, I am able to speak to activities and experiences at Germany’s national public health institute, the Robert Koch Institute (RKI), and to those at the World Health Organization (WHO), where I was within the framework of the West African Ebola outbreak (2013 to 2016) (Owada et al. 2016).

First of all, I agree with Stephen’s analysis that there is the risk, as a result of the successive propagation of algorithmic approaches and technologies for infectious disease control, an epidemiology traditionally based on diagnostic findings and controlled statistical processes is becoming increasingly marginalized and, in parallel, the necessary verification loops are being replaced in favour of ‘Big Data’ ideologies and trends of *Dataification.*^1^ In this context, it seems to me that widespread assumptions that advance the idea that a digital, unofficial infectious disease surveillance and monitoring is quicker than traditional, official information and reporting systems need to be modified. It is true in retrospect that existing digital systems and their associated early warnings could have been faster if their first signals had been correctly named or interpreted at an early stage. At the same time, however, it is mostly ignored that even official state authorities often have knowledge about specific events at relatively early stages – only that they either initially withhold such information or distribute it in other ways according to the official information/notification systems, e.g. the example of Stephen, the authorities of Guinea were aware that there was something going on, but they waited with the reporting. So have I experienced it at the WHO: few countries directly provided all available information to the organization. It can also be observed again and again that official information either minimizes or plays to the media or other entities in a targeted way. In this context, non-state surveillance platforms such as ProMED or HealthMap, for example, should be commended especially for their dimension of political transparency, as they put pressure on governments not to keep information from the public as much as possible. At the same time, however, the increasingly digitized identification, analysis and distribution of epidemiological indications of infectious disease these platforms enable not only leads to increasing likelihood of false positives, but also to specific problems of an immediate, uncontrollable communication of risk. The danger of panic and the great effort required to avoid panic are to be feared.

Henning provides very important information in this regards. He describes that specific public health actors (here: local health authorities) may be compelled to equate technologically-generated signals with epidemiologically certified public health events, and, on the basis of these unproven indications, initiate public health measures. Further, this is also a problematic development from the perspective of resource retention in an already thinly-resourced public health service. Early responses and over-reactions from political decision-makers or the media are to be feared in equal measure. In the broader context of the focus and framework of a ‘Global Health Security’, such potentially exaggerated perceptions and reactions are tied to perceptions of elevated threats of infection – whether from (quasi) natural or man-made infection (e.g., in the context of war or incidents of terror) – and, consequently, to urgent demands for comprehensive and constant attention, outbreak detection, and further crisis/disaster preparedness measures.

### Digital epidemiology and the securitization of Global Health

As a result of this, infectious disease epidemiology is increasingly being, in my humble opinion, in an irritating way integrated into the national and international security architectures. So it was during the West African Ebola outbreak in Sierra Leone and Liberia, two of the three hardest hit countries, that the military was constantly present in the planning of public health measures. E.g. in one situation in Sierra Leone I remember this resulted in prioritizing quarantining over other public health measurements like community engagement. Quarantining is not per se negative but in this particular case turned out very ambivalent as the measure evoked strong resistance among the population and potential new infected individuals increasingly were actively hidden as a consequence. Also in non-outbreak times, the cooperation between security forces and public health entities is becoming increasingly narrow. This can be seen, for example, in the Global Health Security Initiative, which addresses both the biological threats of pandemic flu and possible threats from chemical or radio-nuclear terrorism.

These perspectives as well as the social aspects and subsequent costs of a digitized infection control should be discussed. They are closely linked to the imperatives of constant monitoring and early detection, as well as the similar focus of a ‘Global Health Security’. As such, they should be considered with the view not only to the (not new) anticipated restrictions or marginalizations associated with classical, structural and also socially-reformed, areas of public health, as well as to further possible negative costs resulting from of a ‘securitized’ public health. I would like to hear from you - Stephen and Henning - especially with regard to these broader health and social policy debates, from your social science perspectives, what is your understanding of ‘Global Health Security’ in general and of the ‘preemptive security logic’, which is often discussed in this context, especially?

## Henning

I would suggest to understand “Global Health Security” as a set of preferences and truth claims that are currently framing our understanding of health issues of international relevance. This understanding results from a perspective, that interrogates threat discourses and related policies as a structured but contingent formation of problem descriptions. Problems do not exist ‘naturally’ but they have to be articulated and put on the agenda in a process of social interaction. This approach draws back to Michel Foucault and his proposal to acknowledge a power/knowledge nexus in general and specifically the power effects of truth claims. According to this, articulating and framing an issue are powerful ways to predetermine the range of thinkable approaches and solutions. By using the term “Global Health”, policy-makers, non-governmental actors and academic observers are drawing together several health problems into a common frame, but also marking this frame as a field of intervention and claiming its relevance. The contours of this frame are still blurry and there exist numerous approaches to define “Global Health” (Brown et al. 2006; Farmer et al. 2013; Fassin 2012). There is no accepted definition and “Global health […] is more a bunch of problems than a discipline”. (Kleinman 2010) The ongoing emergence of a problem field “Global health” is an interesting moment then, where new truth claims are put forward and a new understanding of related issues such as ‘health’ and ‘the global’ are formed. Those newly related ideas are powerful as they are confining the agenda setting and plausible goals and methods of intervention.

Approaching “Global health” from this angle, what is striking from the outset is a strong undercurrent of security. The recent surge of “global health” can be attributed to a confluence of two separate discourses. On the one hand, globalization is increasingly narrated as a health risk. An “emerging diseases” discourse paints the picture of a global spread of infectious diseases due to unparalleled levels of global connectivity and frequency of global travel (Barrett et al. 1998). On the other hand, the concept of national security is being reimagined, facing a new multi-polar and complex world order. Today, in order to achieve national security, one has to look beyond military dominance and to take societal issues such as health, poverty but also climate change as security threats into account (Redclift and Grasso 2013). For example in the US, facing the threat of bioterrorism, public health has become a concern for the Department of Homeland Security and international infection control resurfaced as a security issue. Both the fear induced by ‘globalisation of disease’ and the rethinking of national security are underlining a new relevance of global health issues. The resulting tremendous development in global health policies and programs accordingly are often following a security rationale (Genest 2015). One example is the newly installed global health surveillance mechanisms and the revised international health regulations (IHR) (Fidler 2005). The recent conception presents global health as part of a security problem rather than as a humanitarian issue.

This securitization of ‘Global health’ has already been described in some detail (Cook 2010; King 2002; Pereira 2008). Here I want to underline the corresponding shift in the perception of threats and its implications. Current problems of Global health security are often depicted as essentially incalculable. Emerging diseases, acts of intentional Bioterrorism, food security in an increasingly global connected distribution system, antimicrobial resistant agents, − more than ever we now seem to be confronted with “unknown unknowns”. We not only do not know when those events will happen, but we even do not know what the threat is exactly. The reformulated International Health Regulations (IHR) tellingly have shifted from monitoring a fixed catalogue of diseases to the obligation to warn about anything unexpected. According to the IHR, the national health agencies have now to signal any unspecific “public health emergencies of international concern” (World Health Organization 2008) to the WHO. This specific perception of “Global health security problems” as incalculable threats calls for a certain pre-emptive and outbreak-oriented intervention.

The implications of the employed “preemptive security” logic have been detailed in critical security studies (de Goede and Randalls 2009; Lakoff and Collier 2010; Massumi 2007; Caduff 2015). As those studies have shown, preemption often demands the extension of (technological) surveillance and orients efforts towards the event and away from structural conditions. Comparable tendencies have been shown for current “Global health” policies, for example an orientation towards containment of an event rather than the search for a broader structural prevention (Rushton 2011).

To sum it up, I would argue that Global Health is currently presented as a problem and has been put on the political agenda in a way that calls for a very specific answer in the form of a “preemptive security logic”. Firstly, the underlying truth claims about the problems to solve frame the emerging field of Global health partly as security issue. Secondly, the incalculability problem evoked in many threat discourses of current Global health thinking demands a certain security rationale. The problem of an unknown unknown has to be dealt with preemptively. This way of presenting the problem of Global health then implicitly constraints plausible interventions. Approaching health as a security issue does often not tackle the actual problems of health on the ground. For example, this approach inclines to invest scarce resources into monitoring and surveillance rather than education and local health infrastructure. In order to reach the goal of more substantial health policies it is important to be aware of this securitization bias in the current problematization of Global health.

## Stephen

Building further on excellent points articulated by Henning, this epistemic shift in government and politics towards ‘global health security’ has been resultant, as I argue, from significant larger geopolitical transformations, and new reconsiderations of security perspective, in a post-Cold War era of rapidly proliferating *non-traditional security challenges*, which extend beyond traditional security correlations of the state/military, are transnational or global in scope, and again, to underscore the centrality of Henning’s earlier points, which cannot be prevented entirely, only addressed through coping mechanisms and the development of techniques of preemption and forecasting (Caballero-Anthony 2010).

The rise of global health security and its securitizing processes have transformed the ways in which international relations and global politics are understood, orientated and practiced. In 2000 the United Nations Security Council (UNSC) adopted *Resolution 1308* (UNSC 2000) which emphasised that the current HIV/AIDS pandemic, if unchecked, posed a risk to international security and stability, marking the first time in which a health threat was discussed before the UN body mandated to maintain international peace and security (Fidler 2005). 14 years following the seminal Security Council resolution on HIV/AIDS, the United Nations launched its first and only to date, military mission to combat the spread of an infectious disease outbreak. Known as the *United Nations Mission for Ebola Emergency Response* (UNMEER), the first ever UN emergency health mission sought to contain the spiraling West African Ebola outbreak following the UN Security Council Resolution 2177, which determined that the ongoing outbreak in West Africa ‘constituted a threat to international peace and security’ (UNSC 2014), and we can understand these grand transformations within global politics and international relations as permeated by emergent logics to preempt both occurring public health emergencies and also probable future pandemics.

Contrastingly, for critical theorists, global health security has emerged as a concept which denotes a novel biopolitical project, or rather, the appearance of a new *governmental* problem in public health: how to effectively manage ‘emerging infectious diseases’ at a *global* scale (Lakoff 2015). Contemporary global health systems are therefore problematized not only by the rapid emergence of pathogens on a global scale, but the risk posed by these circulating pathogens are no longer calculable using tools of risk assessment, which are based on patterns of historical incidence (ibid). In this regard, global health security rationalities, I assert, galvanise and accelerate the facilitation and development of novel techniques and practices of anticipatory or preemptive security, which emphasise the real-time, continuous and cost-effective surveillance of potential disease outbreak and public health emergencies.

### Digital epidemiology as technologies of preemption

Increasingly, in an era of innumerable digital data sources, the preemption of health risks are managed and analysed via an assemblage of innovative and evolving surveillance practices which combine multiple data sources and disease-tracking techniques, enacted at local, regional and global levels. Syndromic surveillance platforms, and digital epidemic intelligence systems including ProMED-Mail, GPHIN, HealthMap, BioCaster, EpiSPIDER, and the now-defunct Google Flu Trends can thus be conceptualised as new governmental technologies of overarching global health security practices, developed and installed around yet unforeseen events in order to halt or preempt the ‘sudden, circular bolting’ of pandemic phenomena (Foucault 2007).

Collectively then, in my view and building upon the expert points provided by Henning, processes of securitization of global health and the rise of preemptive security logics have advanced calls for the deployment of novel security technologies and surveillance apparatuses over the past two decades. These calls have been met with the re-drawing of disease surveillance operations and the launching of new technologies which now seemingly patrol digital datascapes in the surveillance of potential public health emergencies. Such novel technologies constitute critical components of an evolving ensemble of new governing practices, knowledges, techniques and rationalities of health security, increasingly influenced by digitised, automated and computerized algorithms. .

As components in an emergent socio-technological apparatus of security for the strengthening of global health governmentalities, it is also crucial to consider the ways in which these expanding digital syndromic surveillance systems re-contour previous understandings of the temporalities, form and practice of preemption in the identification of forthcoming pandemics. Firstly, the rise of syndromic surveillance technologies for the forecasting of probable disease outbreaks, departs significantly from previous methodologies to identify and further *preempt* pathogenic threats. As seen with the steady integration of algorithmic programming over the past two decades from ProMED-mail, to GPHIN, and to HealthMap, syndromic surveillance technologies increasingly draw upon and aggregate open-source data pulled via algorithmic processing from the realm of the *digital* to inform contemporary practices of health security in the non-digital/physical world. Within the politics of preemption, this marks a novel transition towards the harnessing of infinite online data sources, afforded by increasingly sophisticated algorithms to identify unusual data correlations or patterns indicative of a potential disease outbreak. In turn, this represents a process that is distinct and divergent from previous methodologies of health surveillance which utilised clinical and laboratory testing, analysis, observation, and the collation of statistics in order to render visible and intelligible, occurring or emergent infectious disease outbreaks. In the new era of digital disease surveillance, the data warehouse emerges alongside the traditional clinic as a new critical site of surveillance and zone of security praxis in the preemption and surveying of disease risk.

Further to this, novel techniques to preempt looming pandemic threats via these digital syndromic surveillance systems now also correspond with new problematizations of data and knowledge forms in the securitization of uncertain [pathogenic] futures. Unlike previous systems of infectious disease surveillance which were routinely marked by an *incompleteness* of data in which to understand forthcoming pandemic risks, the deluge of ‘Big Data’ of the early twenty-first century has now reversed this problematization of data.. Contemporary digital disease surveillance systems and the practice of health security are no longer hindered by a *scarcity* of data but rather *burdened* by an excess of infinitely generating, unstructured and diffuse streams of digital data. In order then to preempt and track the emergence of disease outbreaks in a present world that is submerged in data sources, digital disease practices must navigate, as Matteo Pasquinelli (2015) writes, ‘vast data oceans’ to detect that which constitutes the anomaly, be it common patterns of behaviours in social media, buying or selling tendencies in stock markets, the oscillation of temperatures in a specific region, or suspicious keywords in disease surveillance networks (ibid). Again, in this new practice of ‘navigating vast data oceans’, the digital algorithm emerges once more as a strategic, pragmatic and celebrated technology of government with the capacity to apprehend, process and project new insights of disease patterns from troves of digital data which manifest beyond human cognitive and analytic capacities.

Thus, the politics of preemption in the present era of elevated pandemic threat are intimately intertwined with expanding recourses to apprehending Big Data sources and employing algorithmic processing techniques to produce advanced alerts, indications and insights of potential pathogenic uncertainties.

Indeed, during several critical public health emergencies over the past two decades, a combination of Big Data sources and algorithmic techniques produced meaningful and advanced insights into emergent public health emergencies, including during the early and critical stages of the emergence of severe acute respiratory syndrome (SARS) in China and Ebola in Guinea. However, the success and rise of the algorithm in these health histories should not distract from the imperative for continued meaningful—and indeed critical investigations and interrogations of emergent digital disease surveillance practices which utilize diffuse Big Data sources and processing of such data streams via algorithm.

Algorithms are not only famously opaque, but have also been shown to be cantankerous, if not delicate technologies, illustrated famously by a false reporting of a cholera outbreak in the United States by Google in 2007, as a result of Oprah Winfrey picking *Love in the Time of Cholera* as book of the month in her book club (Simonsen et al. 2016). However, as technology and innovation advance, algorithms are getting smarter, more insightful and more precise, but the growing commonplace of these knowledge producing machines with intensifying technical complexities makes the monitoring and regulation of these data-processing technologies ever the more urgent and vital.

The ascendancy of the era of Big Data and the rise of digital disease surveillance systems have afforded unprecedented new opportunities towards the enhancement and bolstering of disease detection capacities in an era increasingly preoccupied with the emergence of future security challenges—among them pandemic illness. The objective of this discussion has been to provide an overview and highlight the potential gains and benefits yielded by these new data sources and processing techniques, while also emphasising that key ethical, legal, political and societal concerns abound and must not be sidelined in contemporary efforts to accrue maximal data reserves and to effectively track and detect the next pandemic before it occurs.

## Summary

### Tim

Dear Stephen, dear Henning, thank you very much for this inspiring conversation. Again, it made clear the necessity of an interdisciplinary and social sciences inspired debate about contemporary epidemiology and public health.

For me three insights emerge.

First of all, the gains in timeliness and scope of digital epidemiology come at the cost of providing a different type of knowledge. The information provided through such systems is not the same as the traditional expert knowledge based on human assessment, analysis, hypothesis, statistical testing and trials but an algorithmic ‘knowledge without truth’. The status of this knowledge may not be totally clear in all the different contexts where it is used. This may result in ill-informed decision making.

A driving force for the demand of digital epidemiology is a reformulated conception of global health. A common thread running through the diverse debates about global health policies today is the issue of security. This securitization of global health does frame current policies.

Specifically, threats to global health are increasingly identified as incalculable emergencies (unknown unknowns). This results in a demand for preemptive ways to act on those emergencies before they have evolved. This preemptive security logic also fosters an unlimited big data surveillance as a practice of ‘navigating vast data oceans’.

For sure these points need further critical examination. Thus I am looking forward to future interdisciplinary exchange and discussion.

## Footnotes

1 According to Mayer-Schönberger and Cukier (2013) Dataification refers to the transformation of social action into online quantified data, this allowing for the real-time tracking and predictive analysis of events. (Mayer-Schönberger and Cukier 2013)
